# The Efficacy and Safety of Sodium Glucose Cotransporter 2 Inhibitors for Patients with Anticancer Therapy: A Meta-Analysis of Cohort Studies

**DOI:** 10.5334/gh.1440

**Published:** 2025-06-16

**Authors:** Gang Fan, Hong Zuo, Lin Lin, Chao Xu, Rui Yan

**Affiliations:** 1 Cardiology Department of Xianyang Central Hospital, Xianyang, Shaanxi Province, 712000, PR China; 2 Cardiovascular Hospital of the Second Affiliated Hospital of Xi’an Jiaotong University, Xi’an, Shaanxi Province, 710016, PR China; 3 Cardiology Department of Yangling Demonstration District Hospital, Xianyang, Shaanxi Province, 712100, PR China; 4 Cardiology Department of Beijing Luhe Hospital of Capital Medical University, 101149, PR China

**Keywords:** SGLT2 inhibitor, Cancer, Meta-analysis

## Abstract

**Background:**

Chemotherapy-induced cardiotoxicity is the leading cause of non-tumor-related mortality among patients with tumors. Although sodium glucose cotransporter 2 inhibitors (SGLT2is) have been shown to confer cardiovascular benefits, their effects and safety profile in patients with cancer remain uncertain. The objective of this study was to assess the cardiovascular effects of SGLT2is in patients with cancer.

**Objective:**

We conducted a meta-analysis of cohort studies to compare the efficacy and safety of SGLT2is and placebo in patients with cancer.

**Results:**

A total of ten cohort studies, encompassing 85,185 patients, were included in this study. SGLT2is significantly decreased mortality (Risk ratios (RR) 0.52, 95% confidence interval (CI) (0.36, 0.75), I^2^ = 98%), heart failure (HF) (RR 0.43, 95% CI 0.24, 0.77, *I^2^* = 75%), and arrhythmia (RR 0.33, 95% CI 0 .23, 0.49, I^2^ = 0%). In addition, SGLT2is decreased the incidence of adverse events. No significant difference was identified in hypoglycemia, ketoacidosis, and acute coronary syndrome (ACS).

**Conclusion:**

The present study suggest that sodium glucose cotransporter 2 inhibitors may be an efficacious and safe means for improving the prognosis of patients with cancer and diabetes. However, future large-scale randomized controlled trials are needed to further validate the results.

## Introduction

Cancer represents the second most common cause of mortality in the United States overall and the primary cause of death among individuals under the age of 85 (1). The relative five-year survival rate for all cancers combined has increased from 49% for cases diagnosed during the mid-1970s to 69% for those diagnosed during 2013–2019 (2). The survival rate of patients with cancer is on the rise because of advancements in early cancer detection and treatment. The adverse effects of anticancer pharmacotherapy on the cardiovascular system can manifest in several pathological ways, including cardiomyopathy, hypertension, thromboembolism, aberrant conduction, and metabolic disorders collectively referred to as cancer therapy-related cardiovascular toxicity (CTR-CVT) (3). CTR-CVT has become a common challenge encountered by both patients and clinicians (4).

Cardiovascular disease (CVD) has emerged as the second leading cause of morbidity and mortality in cancer survivors (5). The early diagnosis of CTR-CVT and the prompt initiation of neurohormonal therapy have been associated with an optimal clinical prognosis. The use of established pharmacological agents, including sacubitril valsartan (6), beta-blockers (7), and dexrazoxane (8) has demonstrated some efficacy in reducing CTR-CVT. Nevertheless, the overall benefit-to-risk ratio remains suboptimal, and the incidence of morbidity and mortality remains high among patients with cancer.

Sodium-glucose cotransporter-2 (SGLT2) inhibitors originally developed with the objective of improving the management of type 2 diabetes mellitus (T2DM) by inhibiting glucose reabsorption in the renal proximal tubules (9). In addition to their glucose-lowering effects, extensive studies have demonstrated that SGLT2 inhibitors possess a range of other beneficial properties (10,11). It has been proven that SGLT2 inhibitors display exceptional cardioprotective characteristics in patients, regardless of their diabetic status (12). Moreover, recent studies have indicated the potential for SGLT2 inhibitors administered in advance to confer cardioprotective benefits in patients with cancer (13,14). Notwithstanding these findings, there is currently a paucity of studies examining the protective role of SGLT2 inhibitors against CTR-CVT. Furthermore, there are no randomized controlled trials (RCT) studies that have been published, and the specific role and mechanisms remain unclear. Considering the circumstances, we conducted a meta-analysis with the aim of providing more detailed information for future clinical applications of SGLT2 inhibitors with the goal of optimizing the prognoses.

## Methods

This study was conducted in accordance with the Preferred Reporting Items for Systematic Reviews and Meta-Analyses (PRISMA) guidelines (15). As the investigation was based on publicly available data, ethical approval and patient consent were not required.

### Search strategy

A systematic search of the PubMed, Cochrane Library, and Embase databases from inception to August 2024 was conducted to identify cohort studies that directly compared the CTR-CVT in cancer patients. The following terms were used in the search: SGLT2, Cancer, Empagliflozin, Dapagliflozin, Ertugliflozin, Canagliflozin, Sotagliflozin, Anthracyclines, Malignancy, Cardiotoxicity, and Cardiovascular outcomes.

### Inclusion criteria

Studies were included based on the following criteria: 1) retrospective or prospective cohort studies; 2) patients with cancer undergoing chemotherapy or radiotherapy; 3) intervention groups where SGLT2 inhibitors were administered; 4) reported data encompassing at least one of the following outcomes: mortality, heart failure, arrhythmia, or adverse events; and 5) follow-up durations not less than three months.

### Exclusion criteria

Studies were excluded based on the following criteria: 1) not cohort studies or follow-up studies less than three months; 2) meta-analysis or review studies; 3) ongoing or unpublished studies; 4) studies not published in English; 5) studies where data is either incalculable or not original; and 6) patients with baseline cardiovascular disease.

### Clinical outcome

The principal endpoints were changes in mortality and heart failure. The secondary endpoints included the incidence of arrhythmia, sepsis, urinary tract infection and other safety outcomes.

### Data extraction and quality assessment

Two independent reviewers (Gang Fan and Chao Xu) employed a predesigned form to extract data from the included studies. The extracted information included the first author, country, journal, year of publication, age of participants, number of participants, type of cancer therapy, and follow-up period. Any inconsistencies were addressed through a process of discussion with a third reviewer (Lin Lin). The quality of the included cohort studies was evaluated using the Newcastle-Ottawa Scale (NOS) (16). The scale assesses three domains: the selection of study groups, the comparability of groups, and the ascertainment of outcomes. A score of 7 or above was deemed to indicate a high-quality study.

### Statistical analysis

The data were analyzed by the Meta package of R software. The effects and safety profile of SGLT2 inhibitors in cancer patients were assessed by pooled risk ratio (RR) with 95% confidence interval (CI). The degree of heterogeneity was evaluated using the Cochran’s Q test and quantified through the I² statistic. And I² value of 25%, 50%, or 75% was deemed to indicate low, moderate, or high heterogeneity, respectively. In instances where heterogeneity was low or moderate, a fixed-effect model was employed. Conversely, when heterogeneity was high, a random-effect model was utilized for the synthesis of results from included studies. Furthermore, the use of the prediction interval (PI) is recommended when actual heterogeneity is anticipated, as the PI is regarded as a more conservative approach to incorporating uncertainty into studies. To address the issue of heterogeneity, we conducted a series of subgroup and sensitivity analyses. Meanwhile, publication bias was evaluated using funnel plots and the Egger test and Begg test. A statistically significant difference was defined as *P* < 0.05.

## Results

### Included studies and quality assessment

**Figure 1:**
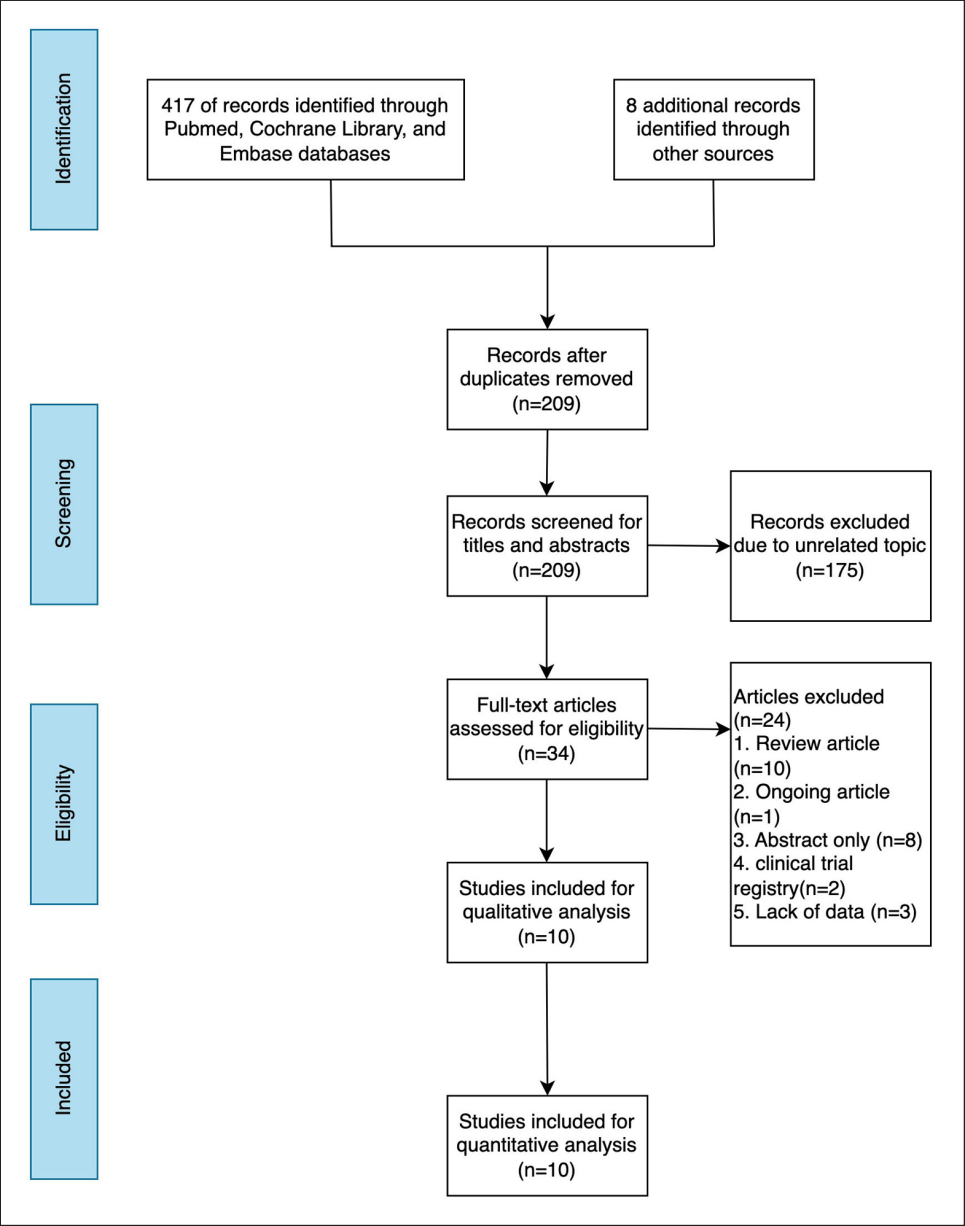
The flowchart for the study.

In the initial searches, we found 425 possible qualifying references. Following the removal of duplicate articles, a total of 209 appropriate references were subjected to screening. Based on the titles and abstracts, 175 records were excluded. Of the remaining 34 publications, 10 were review articles, 1 was an ongoing study, 8 were only abstracts, 2 were clinical trial registries, and 3 studies were excluded due to a lack of data. In the end, our meta-analysis included 10 eligible studies (13,14,17,18,19,20,21,22,23,24). The flowchart for the study is presented in (Figure 1).

The baseline characteristics of the studies included in the analysis are presented in (Table 1). Among the included studies, six were conducted in the USA, and the other four studies were conducted in Canada, Israel, China (Taiwan), and South Korea. A total of 85,185 patients were included in this study, comprising 20,538 individuals in the SGLT2 inhibitors group and 64,647 in the control group. The follow-up period spanned a range of 18 to 57.6 months. The patient cohort was aged between 56 and 77 years. All the included studies were of a high quality, meeting the Cochrane criteria for parallel group research.

**Table 1:** Characteristics of included studies and populations.


Study	Country	Study Design	Journal	Participants (n)		Age (mean SD)		Male (%)		T2DM	Chemotherapy	Cancer type	Follow-up (Months)	NOS

				SGLT2i	Control	SGLT2i	Control	SGLT2i	Control					

Gongora 2022	USA	Retrospective cohort study	JACC: Heart Failure	32	96	60 (11)	60 (10)	50	57	Yes	Anthracyclines	Lymphoma, Breast cancer, Leukemia, Genitourinary,Gastrointestinal and other cancers	18	8

Perelman 2024	Israel	Retrospective cohort study	Cardio-oncology	24	95	70 (6)	71 (11)	79	58	Yes	Immune checkpoint inhibitors	NSCLC, Melanoma, Renal cell carcinoma, Hepatocellular carcinoma, Breast cancer, Cenical Squamous, Other cancers	28	7

Fath 2024	USA	Retrospective cohort study	The American Journal of Cardiology	706	706	62.5 (10.1)	62.4 (12.7)	48	47	Yes	Anthracyclines	Hematological and lymphatic, Breast, Gastrointestinal and digestive organs, Respiratory and intrathoracic organs, Endocrine glands and other cancers	24	9

Avula 2023	USA	Retrospective cohort study	JACC: Heart Failure	640	640	67.6 (10.8)	67.6 (11.6)	58.4	58.4	Yes	Anthracyclines, Antimetabolites. Aromatase inhibitor, monoclonal antibodies, Proteosome inhibitors, Small molecular TKIs	Breast, Lymphomas, Myelodysplastic syndromes, Genitourinary,Gastrointestinal, Gynecologic and other cancers	24	9

Qadir 2023	Canada	Retrospective cohort study	JACC: Cardiooncology	99	834	NS	NS	35.4	38.1	Yes	Anthracyclines	Breast cancer, Lymphoma, other maligancy	19.2	9

Chiang 2022	USA	Retrospective cohort study	Heart	878	878	NS	NS	54	52	Yes	Antimetabolites, Platinum, Plant alkaloids, Anthracyclines, Tyrosine kinase inhibitors, HER2 inhibitors, Immune checkpoint inhibitor	Gastrointestinal, Genitourinary, Thoracic, Head and Neck, Breas, Hematologic, Skin, Other cancers	18.8	9

Hwang 2023	South Korea	Retrospective cohort study	Scientific Reports	779	2337	56 (10)	62 (11)	29	37	Yes	Anthracyclines, Alkylating agents, HER2 inhibitor, VEGF targeting agents	Lymphoma, Breast cancer, Genitourinary, and other cancers	40.8	7

Huang 2024	China (Taiwan)	Retrospective cohort study	Diabetes & Metabolism	16711	33422	61 (110	62 (11)	48	48	Yes	Not specified	Pancreatic cancer, Hepatocellular carcinoma, Esophageal, head and neck, lung and other cancers	57.6	9

Luo 2023	USA	Retrospective cohort study	British Journal of Cancer	531	24384	73.5 (5.45)	77 (6.73)	54.24	50.82	Yes	Not specified	Non-small cell lung cancer	21.2	9

Hendryx 2022	USA	Retrospective cohort study	Plos One	137	137	72.5 (5.20)	74.9 (6.52)	68.61	68.27	Yes	Not specified	Hepatocellular carcinoma	20.4	9


### Comparison of mortality between groups

**Figure 2:**
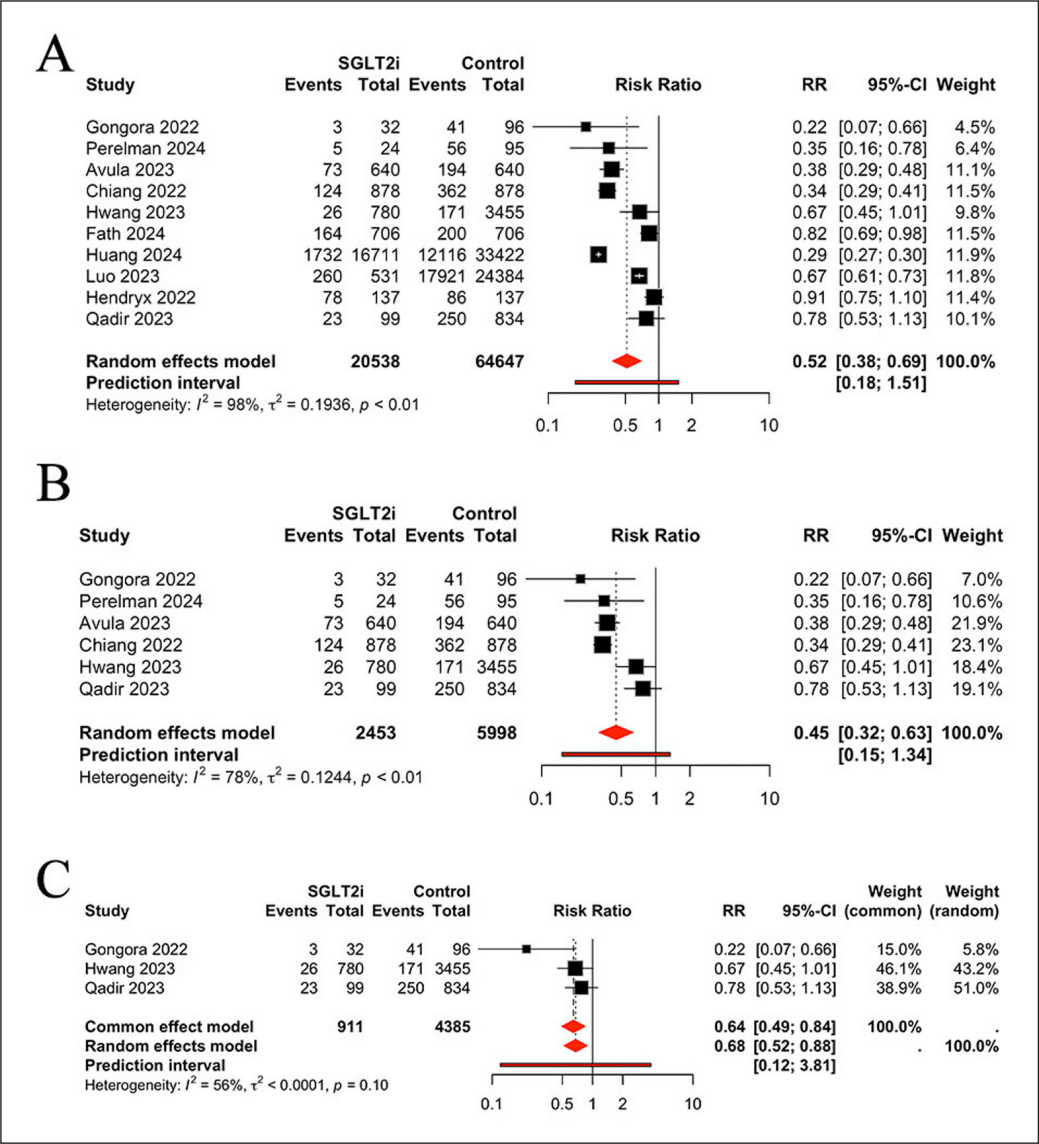
Comparison of mortality between groups, **A:** Total analysis; **B:** Sensitivity analysis. **C:** Results limited to anthracycline-based chemotherapy.

A total of 10 studies were identified that reported mortality rates. As shown in Figure 2A, SGLT2 inhibitors application significantly reduced mortality compared to the control group (RR 0.52, 95% CI 0.38, 0.69, *P* < 0.0001, I^2^ = 98%, PI 0.18, 1.51). Given the considerable heterogeneity between studies (I² = 98%), we conducted a sensitivity analysis. The results indicated that SGLT2 inhibitors remain effective (RR 0.45, 95% CI 0.32, 0.63, *P* < 0.0001), with moderate heterogeneity (I² = 78%) (Figure 2B). Further subgroup analysis, limited to studies utilizing anthracycline-based chemotherapy, revealed that the mortality benefit remained statistically significant (RR 0.68, 95% CI 0.52, 0.88, *P* < 0.0001), also with moderate heterogeneity (I² = 56%) (Figure 2C).

### Comparison of heart failure between groups

**Figure 3:**
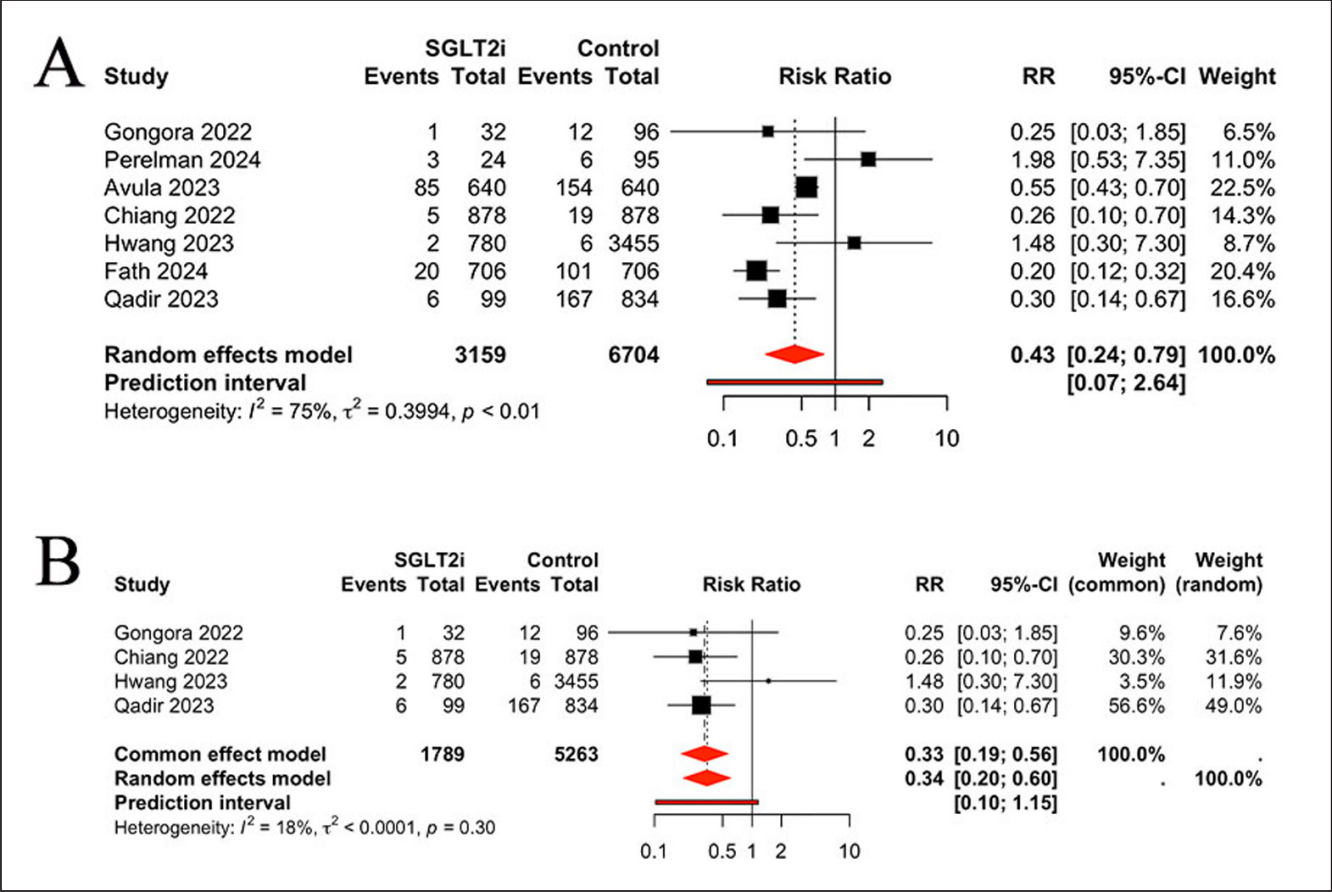
A: Comparison of heart failure between groups; **B:** Sensitivity analysis.

Seven studies were identified for the purpose of evaluating heart failure between the two groups. Patients who used the SGLT2 inhibitors had a significantly lower risk of heart failure than those without SGLT2 inhibitors (RR 0.43, 95% CI 0.24, 0.79, *P* = 0.0063, I^2^ = 75%, PI 0.07, 2.64) (Figure 3A). Considering the moderate heterogeneity between studies (I² = 75%), a sensitivity analysis was conducted. The results indicated that SGLT2 inhibitors still effective (RR 0.33, 95% CI 0.19, 0.56, *P* < 0.0001), with low heterogeneity (I² = 18%) (Figure 3B).

### Comparison of arrhythmia and ACS (Acute coronary syndrome) between groups

**Figure 4:**
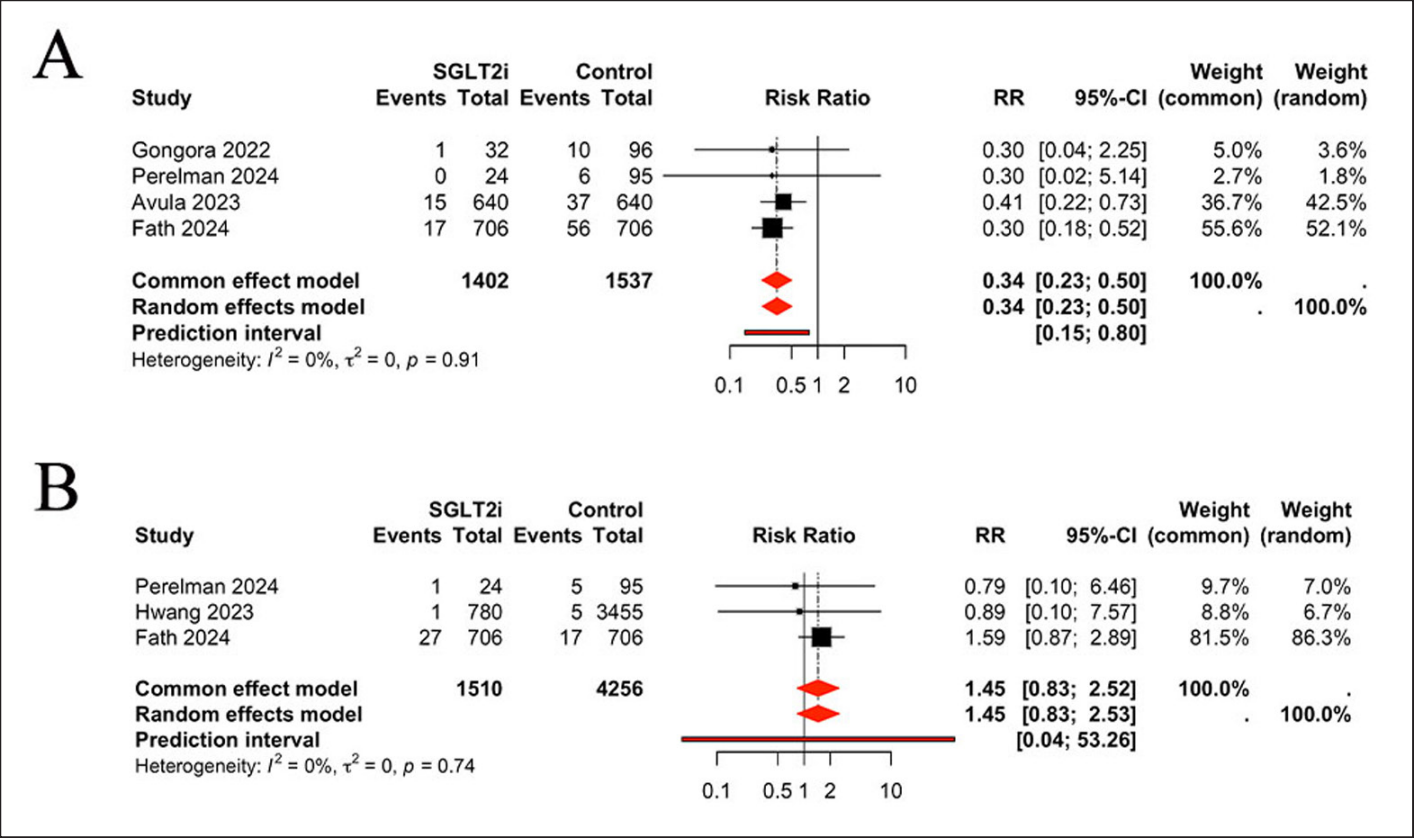
Comparison of arrhythmia **(A)** and ACS **(B)** between groups, ACS: acute coronary syndrome.

Four of the included studies reported the occurrence of arrhythmia in patients undergoing treatment with SGLT2 inhibitors. The pooled results demonstrated a reduction in the incidence of arrhythmia in patients treated with SGLT2 inhibitors, in comparison to the control group (RR 0.34, 95% CI 0.23, 0.50, *P* < 0.0001, I^2^ = 0%) (Figure 4A). Three studies were identified that reported the occurrence of ACS. No significant differences were observed between the SGLT2 inhibitors and control groups regarding the incidence of ACS (RR 1.45, 95% CI 0.83, 2.52, *P* > 0.05, I^2^ = 0%) (Figure 4B).

### Comparison of safety outcomes between groups

**Figure 5:**
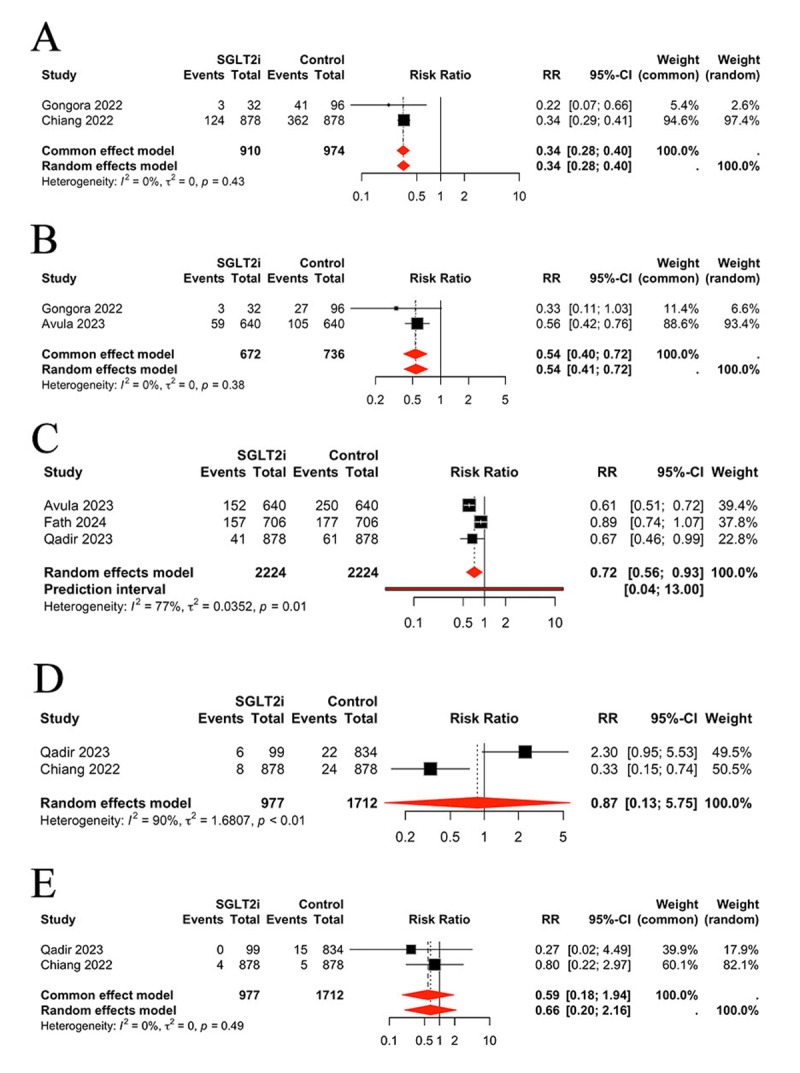
Comparison of safety outcomes between groups, **A:** Comparison of sepsis between groups, **B:** Comparison of urinary tract infection between groups, **C:** Comparison of kidney injury between groups, **D:** Comparison of hypoglycemia between groups, **E:** Comparison of ketoacidosis between groups.

In the final analysis, we assessed the safety outcomes associated with the SGLT2 inhibitor intervention. The results indicated that SGLT2 inhibitors decreased the incidence of adverse events such as sepsis (RR 0.34, 95% (CI 0.28, 0.40), *P* < 0.0001, I^2^ = 0%) (Figure 5A), urinary tract infection (UTI) (RR 0.54, 95% CI (0.40, 0.72), *P* < 0.0001, I^2^ = 0%) (Figure 5B), and kidney injury (RR 0.72, 95% CI (0.56, 0.93), *P* = 0.0104, I^2^ = 0%) (Figure 5C). No significant difference was identified in hypoglycemia (RR 0.87, 95% CI (0.13, 5.75), *P* > 0.05, I^2^ = 90%) (Figure 5D) or ketoacidosis (RR 0.59, 95% CI (0.18, 1.94), *P* > 0.05, I^2^ = 0%) (Figure 5E).

### Comparison of hospitalization between groups

Three studies were identified for the purpose of assessing hospitalization between the two groups. The hospitalization rate between the two groups was not significantly different (RR 0.46, 95% CI 0.20, 1.02, *P* = 0.09, I^2^ = 58.8%, PI 0.02, 8.49) (**Supplementary Figure S1**).

### Publication bias

The funnel plot of mortality outcomes exhibited an asymmetric distribution, indicating the potential for publication bias (**Supplementary Figure S2**). The funnel plot of heart failure outcomes displayed a slight asymmetric data distribution, suggesting the possibility of publication bias (**Supplementary Figure S3**). Nevertheless, the Egger and Begg tests (*P* > 0.05) and sensitivity analysis demonstrated that the results were consistent with the primary random-effects analysis. A Trim and Fill analysis was also performed, with three supplementary studies incorporated into the analysis of mortality. The results indicated that SGLT2 inhibitors application significantly reduced mortality compared to the control group (RR 0.37, 95% CI 0.25, 0.55, *P* < 0.0001, thereby providing further corroboration for the stability of the primary results (**Figure S4**). The Trim and Fill analysis for heart failure added no additional studies and the results showed the same results as the primary analysis.

## Discussion

The present study included 10 eligible studies with an NOS score above 7, which indicates their high quality, and the efficacy and safety of SGLT2 inhibitors in patients with cancer was assessed. We demonstrate for the first time that SGLT2 inhibitors reduce the incidence of mortality, heart failure, and arrhythmia without any safety concerns in patients with cancer and diabetes.

The convergence of cancer and CVD represents a rapidly expanding patient population with unique and specific requirements in terms of healthcare provision (25). Cancer and CVD are the most significant contributors to global morbidity and mortality from non-communicable diseases. Anthracyclines have a broad range of applications in the treatment of solid tumors and hematological cancers. A dose-dependent relationship has been observed between the administration of these agents and the subsequent development of treatment-related left ventricular (LV) compromise (26,27). Heart failure also represents one of the most significant long-term adverse effects of anthracyclines therapy observed in childhood cancer survivors, with a notable association with increased mortality (28). Epidemiological studies have demonstrated that cardiomyopathy caused by anthracycline antineoplastic drugs is present in up to 57% of cases (29). It is also recognized that anticancer therapy can result in the induction of arrhythmia, including bradycardia (30), atrial fibrillation (31), ventricular tachycardia (32), and corrected QT interval prolongation (33). It is recommended that a baseline assessment of cardiac function be conducted on all patients prior to the initiation of any potentially cardiotoxic therapy, as this may help to reduce the incidence of CTR-CVT (34). Previous studies have indicated that a few CVD medications have a preventive effect against CTR-CVT. These include β-blockers, angiotensin-converting enzyme inhibitors, angiotensin-receptor blockers, spironolactone and statin (35,36). Also, the concomitant use of dexrazoxane, which was initially developed as an iron chelator, has also been shown to have cardioprotective effects in cancer patients through its interaction with topoisomerase IIβ (37). However, the evidence for this is not entirely conclusive.

A study from Taiwan (China) indicates that SGLT2 inhibitor dapagliflozin protects doxorubicin-induced cardiotoxicity in patients with breast cancer by reducing apoptosis and diminishing the expression of endoplasmic reticulum (ER) stress-associated proteins, including glucose-regulated protein 78 (GRP78), protein kinase RNA-like endoplasmic reticulum kinase (PERK), eukaryotic translation initiation factor 2 alpha (eIF-2α), activating transcription factor 4 (ATF-4), and C/EBP-homologous protein (CHOP) (38). Another study found that empagliflozin improve cardiac function in doxorubicin treated mice by inhibiting ferroptosis, fibrosis, apoptosis and inflammation (39). The results of our study are consistent with the above animal studies. We found that SGLT2is significantly decreased mortality (RR 0.52, 95% CI 0.38, 0.69, *P* < 0.0001, I^2^ = 98%), and heart failure (HF) (RR 0.43, 95% CI 0.24, 0.79, *P* = 0.0063, I^2^ = 75%). A prior investigation revealed that SGLT2 is were associated with a reduced risk of all-cause mortality in patients with diabetes and cancer (RR 0.46, 95% CI 0.31–0.68). The present study offers further substantiation of the effects of SGLT2 on mortality and provides new evidence of its effects on ACS, arrhythmia, UTI, kidney injury, hypoglycemia, sepsis and other indices (40). In another study, three cohort studies were included, and only the effect of SGLT2is on anthracycline-induced cardiotoxicity was analyzed (41). Our study not only corroborated some of the conclusions of the aforementioned studies but also provided novel evidence that lends further support to the management of patients with diabetes and cancer with SGLT2is. A meta-analysis comprising 52,115 patients demonstrated that SGLT2 inhibitors are associated with a reduction in the incidence of cardiac arrhythmia (42). A further meta-analysis involving 63,116 patients determined that SGLT2 inhibitors decrease the risk of incident atrial arrhythmias and sudden cardiac death in patients with T2DM (43). Our study represents the inaugural demonstration that SGLT2 inhibitors reduce arrhythmias caused by oncology treatment arrythmia (RR 0.34, 95% CI 0.23, 0.50, *P* < 0.0001, I^2^ = 70%). Chemotherapy-induced arrhythmia constitutes a significant potential complication of treatment, with the potential to markedly increase morbidity and mortality (44). The present study thus offers a significant reference point for the use of SGLT2 inhibitors in the prevention and treatment of arrhythmias associated with tumor therapy.

The published cohort studied indicated that the incidence of SGLT2 inhibitor use among older women and men is associated with an elevated risk of genital mycotic infections within 30 days. However, there is no associated increased risk of UTI (45,46). A real-world study revealed that, irrespective of age, sex, prior-existing cardiovascular disease, or type of SGLT2 inhibitor used, patients with type 2 diabetes who were initiated on SGLT2 inhibitors exhibited a lower incidence of sepsis risk (47). The results of our study showed that SGLT2 inhibitors were safe in cancer patients. We found that SGLT2 inhibitors decreased the incidence of sepsis (RR 0.34, 95% CI 0.28, 0.40, *P* < 0.0001, I^2^ = 0%), UTI (RR 0.54, 95% CI 0.40, 0.72, *P* < 0.0001, I^2^ = 0%), and kidney injury (RR 0.72, 95% CI 0.56, 0.93, *P* = 0.0104, I^2^ = 0%).

The precise mechanism through which SGLT2 inhibitors exert a protective role in CTR-CVT may encompass the reduction of oxidative stress, inflammation and apoptosis, in addition to enhancements in mitochondrial function and cardiac energy metabolism (48,49,50). In addition, it was demonstrated that empagliflozin induced a metabolic shift in the rat heart, resulting in reduced glucose oxidation and enhanced utilization of ketones. This led to improved cardiac function and energetics during both ischemia and reperfusion (51). Finally, preclinical studies have demonstrated that SGLT2 inhibitors exert direct anti-tumor effects, which may offer a potential dual benefit in patients diagnosed with cancer (52,53).

It is important to note that chemotherapeutic agents have the capacity to affect any element of the nephron, which can result in a range of serious health consequences, including acute and chronic interstitial nephritis, as well as acute kidney injury (54). The osmotic diuretic effect of SGLT2 has the potential to increase the nephrotoxicity, ketoacidosis and hyperglycemia of antineoplastic agents (55). However, our study did not observe significant difference regarding hypoglycemia (RR 0.87, 95% CI (0.13, 5.75), *P* > 0.05, I^2^ = 90%) or ketoacidosis (RR 0.59, 95% CI (0.18, 1.94), *P* > 0.05, I^2^ = 0%). In the contrary, we determined that SGLT2is reduce UTI and kidney injury.

### Limitations

1) In view of the paucity of available data, the present study comprised patients with diabetes undergoing anticancer therapy. Consequently, further investigation is required to draw conclusions regarding nondiabetic cancer patients. 2) The present study indicated that SGLT2is has the potential to significantly improve the prognosis of patients diagnosed with cancer. However, it should be noted that the clinical setting, the duration of SGLT2 inhibitor medication use, and the specific type of cancer varied considerably across the included studies. 3) It is imperative that future large-scale randomized controlled trials are conducted to validate the results of this study.

## Conclusions

The present study indicates that sodium glucose cotransporter 2 inhibitors are an efficacious and safe means for improving the prognosis of patients with cancer and diabetes. Nevertheless, future studies are needed to further verify the findings of this study.

## Additional File

The additional file for this article can be found as follows:

10.5334/gh.1440.s1 Supplemental files. Figures S1 to S4 and Table S1.

## Data Accessibility Statement

Data will be made available on reasonable request.

The data used to support the findings of this study are included within the article. Data are available from the corresponding author.

## Author contricutions

Gang Fan and Rui Yan conceived and designed the study; carried out the literature search, study selection, and quality assessment; finished data extraction; confirmed the authenticity of all the raw data; performed the statistical analysis; and wrote the manuscript. Lin Lin, Hong Zuo, and Chao Xu carried out the literature search, study selection, and quality assessment. Lin Lin, Chao Xu, and Hong Zuo finished data extraction, confirmed the authenticity of all the raw data, interpreted the data, and revised the manuscript. All authors have read and approved the final manuscript.
